# The Achilles tendon Total Rupture Score is a responsive primary outcome measure: an evaluation of the Dutch version including minimally important change

**DOI:** 10.1007/s00167-020-05924-7

**Published:** 2020-03-07

**Authors:** Olivier C. Dams, Inge H. F. Reininga, Johannes Zwerver, Ronald L. Diercks, Inge van den Akker-Scheek

**Affiliations:** 1 Department of Orthopaedic Surgery, University of Groningen, University Medical Center Groningen, Hanzeplein 1, 9713 GZ Groningen, The Netherlands; 2 Department of Trauma Surgery, University of Groningen, University Medical Center Groningen, Hanzeplein 1, 9713 GZ Groningen, The Netherlands; 3 Department of Human Movement Sciences, University of Groningen, University Medical Center Groningen, Hanzeplein 1, 9713 GZ Groningen, The Netherlands; 4 Sports Valley, Gelderse Vallei Hospital, Willy Brandtlaan 10, 6716 RP Ede, The Netherlands

**Keywords:** Achilles tendon rupture, PROM, Questionnaire, Epidemiology, ATRS, Dutch, Responsiveness, Minimally important change, Follow-up

## Abstract

**Purpose:**

Aim of this study was to evaluate the responsiveness of the Dutch version of the Achilles tendon Total Rupture Score (ATRS-NL).

**Methods:**

Patients (*N* = 47) completed the ATRS-NL at 3 and 6 months after Achilles tendon rupture (ATR). Additionally, they filled out the Euroqol-5D-5L (EQ-5D-5L) and Global Rating of Change Score (GRoC). Effect sizes (ES) and standardized response means (SRM) were calculated. The anchor-based method for determining the minimally important change (MIC) was used. GRoC and improvement on the items *mobility* and *usual activities* on the EQ-5D-5L served as external criteria. The scores on these anchors were used to categorize patients’ physical functioning as *improved* or *unchanged* between 3 and 6 months after ATR. Receiver operating curve (ROC) analysis was performed, with the calculation of the area under the ROC curve (AUC) and the estimation of MIC values using the optimal cut-off points.

**Results:**

There was a large change (ES: 1.58) and good responsiveness (SRM: 1.19) of the ATRS-NL between 3 and 6 months after ATR. Using ROC analysis, the MIC values ranged from 13.5 to 28.5 for reporting improvement on EQ-5D-5L *mobility* and GRoC, respectively. The AUC of improvement on mobility and improvement on GRoC were > 0.70.

**Conclusion:**

The ATRS-NL showed good responsiveness in ATR patients between 3 and 6 months after injury. Use of this questionnaire is recommended in clinical follow-up and longitudinal research of ATR patients. MIC values of 13.5 and 28.5 are recommended to consider ATR patients as improved and greatly improved between 3 and 6 months after ATR.

**Level of evidence:**

II.

## Introduction

Patient-reported outcome measures (PROMs) provide the patient’s perception of the recovery and outcome of treatment [1]. To be applicable and useful in research, PROMs must show adequate measurement properties [2]. Traditionally, validity and reliability were considered the fundamental characteristics of a measurement instrument [3]; while these provide sufficient information on an instrument’s descriptive properties, responsiveness is required before it can be used as an outcome measure to assess change over time [4–6]. Responsiveness is defined as ‘the ability of an instrument to detect changes over time’ [7]. Without evidence that the PROM is sensitive to changes over time, usefulness in longitudinal research is insufficient, as meaningful effects go undetected [8]. PROMs are also increasingly used to enhance clinical management and to evaluate the results of treatment over time [1, 9]. Given that the primary goal of medical treatment is to produce change, responsiveness is also an essential property for clinicians choosing a PROM for patient follow-up [6, 10].

Several PROMs are available to assess outcome in foot and ankle and Achilles tendon disorders [11, 12]. To assess the outcome after Achilles tendon rupture (ATR) specifically, only one tool exists that is considered valid and reliable: the Achilles tendon Total Rupture Score (ATRS) [13]. This 10-item questionnaire has been translated and shows sufficient validity and reliability in several languages [14–24], including Dutch (ATRS-NL) [25]. However, data on the responsiveness of the ATRS-NL are lacking.

Overall, there are two methods to assess the responsiveness of a PROM: a distribution-based and an anchor-based method. Besides the study regarding the development and validation of the ATRS [26], only three studies have assessed the responsiveness of a translation of the ATRS, determining effect size (ES) and relative efficiency using distribution-based approaches [17, 18, 27]. Distribution-based approaches assess change based on the statistical characteristics of the sample. A limitation of distribution-based approaches is that they do not provide data on clinically relevant change [28–30]. Using anchor-based approaches, the smallest change in score that is considered a relevant change by either the patient or the clinician, the minimally important change (MIC), can be calculated [7]. These data facilitate the interpretation of change scores and increase the usability of PROMs [31]. To our knowledge, there are no data on the MIC of the ATRS questionnaire in any language. The aim of this study was therefore to investigate the responsiveness of the ATRS-NL.

## Materials and methods

Data for this study were collected from a study population of a multicentre prospective cohort study. The study protocol has been previously described [32]. All participants that participated in the 3-month (T1) and 6-month (T2) follow-up measurements were included in the current study. This study was approved by the local Medical Ethical Committee (METc) of the University Medical Center Groningen (UMCG) (METc 2017/126). This study was locally approved (local feasibility) by the Medical Ethical Committees of the Martini Hospital Groningen (MHG) (MEC 2017–087) and Medical Center Leeuwarden (MCL) (COV 274(a)).

### Participants

A multicentre prospective cohort study was performed at the UMCG, MHG and MCL in the Netherlands between July 2017 and May 2019. Inclusion criteria for this study were patients aged 18 or older who were diagnosed and treated for an acute total ATR at UMCG, MHG, and/or MCL. Participants were included within the first 3 months post-injury. Exclusion criteria were inability to read Dutch or cognitively understand the questionnaires. All participants were given written and oral information prior to granting informed consent.

A total of 50 participants were included in the multicentre prospective cohort study, three of whom were lost to follow-up between T1 and T2. This study, therefore, included a total of 47 participants who had measurements at both T1 and T2.

### Outcome measures

#### Dutch version of the Achilles tendon Total Rupture Score (ATRS-NL)

The ATRS-NL is a disease-specific, self-administered PROM that can be used to measure outcome related to symptoms and physical activity after treatment in patients with an ATR [25, 26]. This PROM consists of 10 questions using an 11-point (0–10) Likert scale, with a maximum score (= maximum disability) of 100 points. A minimum score of 0 indicates no symptoms and full function/recovery. Five questions address symptoms and five questions address physical activity related to ATR [26]. This instrument is considered a valid and reliable method to evaluate outcome in ATR patients [25, 26].

#### Dutch version of the Euroqol-5D-5L (EQ-5D-5L)

The EQ-5D-5L is a commonly used generic questionnaire to measure health-related quality of life. It encompasses five subdivisions related to physical, mental, emotional and social functioning where, in the 5L-version, questions are administered on a 5-point Likert scale pertaining to the five levels/dimensions of severity: no problems (1), slight problems (2), moderate problems (3), severe problems (4) and extreme problems (5) [33].

#### Anchors

No gold-standard external criterion for improvement (after ATR) exists, therefore it is recommended to use multiple independent anchors [34–37]. Both global ratings and longitudinal disease-related measures of outcome are recommended for determining meaningful clinical change [34]. This study, therefore, used three patient-reported anchors as external criteria: the Global rating of Change score (GRoC) and the EQ-5D-5L items *mobility* and *usual activities*.

The GRoC was constructed based on the methods of Jaeschke et al. [38]. It can quantify the degree in patient-perceived improvement over a specified period of time [38]. Participants were asked to report the perceived change in symptoms and impairment regarding the injured Achilles tendon at 6 months compared to 3 months after ATR. The magnitude of this change was scored on a 5-point Likert scale ranging from ‘much more impairment’ (− 2), to ‘more impairment’ (− 1) ‘about the same’ (0), ‘less impairment’ (+ 1) and ‘much less impairment’ (+ 2).

Two other anchors were derived from the Dutch version of the EQ-5D-5L. Because the ATRS was developed and found valid to measure outcome related to symptoms and physical activity, this study used the EQ-5D-5L items related to mobility and usual activities. An evaluation of responsiveness of the English ATRS also used these subdivisions [27].

### Study procedures

At T1 ATR patients were administered the ATRS-NL and the Dutch version of the EQ-5D-5L. Three months later (T2) participants were administered both these questionnaires for the second time in combination with the GRoC.

### Data analysis

The scores on the GRoC scale were dichotomized. Participants who reported ‘much less impairment’ (+ 2) were categorized as *improved.* Similarly to other researchers, participants reporting ‘less impairment’ (+ 1) were classified as equivalent to ‘about the same’ (0) for the MIC analysis [39] and categorized as *unchanged*. Participants reporting ‘more impairment’ (− 1) and ‘much more impairment’ (− 2) were excluded from the anchor-based MIC analysis.

The change in scores on the EQ-5D-5L subdivisions *mobility* and *usual activities* were also dichotomized. Participants reporting an increase in at least one dimension (≥ 1) were considered *improved* and those reporting no increase in dimensions (0) were considered *unchanged*. Participants reporting a decrease in at least one dimension were excluded from the anchor-based MIC analysis.

For the ATRS-NL to be responsive it needs to demonstrate a lack of floor and ceiling effects, meaning participants should not record the maximum or the minimum score for each time point. Floor and ceiling effects were present if more than 15% of respondents achieved the lowest or highest possible scores [30].

### Statistical analysis

All statistical analyses were performed using IBM SPSS software, version 23.0 for Windows (IBM Corporation, Armonk, NY). A *p* value < 0.05 was considered statistically significant in all analyses. Descriptive statistics (mean and standard deviation (SD), median and interquartile range (IQR), and frequencies) were used for participant characteristics and to display outcomes of the questionnaires. Both distribution-based and anchor-based methods were applied to assess responsiveness.

#### Distribution-based approach

The effect sizes (ES) as described by Cohen et al. were calculated [40, 41]. Using this method the difference between the mean ATRS-NL T2 and T1 scores, divided by the SD of T1 scores was calculated. A value of 0.2 represents a small change (one-fifth of the baseline SD), 0.5 a moderate change and > 0.8 a large change in score.

The standardized response mean (SRM) was calculated according to the method described by Liang et al. [42]. This measure compares the results of the ATRS-NL scores at T1 and T2 and examines the magnitude of the change in scores. The SRM is the ratio of the mean change between T2 and T1 to the SD of this mean change [29, 42]. SRM values < 0.5 are considered to indicate low responsiveness, 0.5–0.8 moderate and > 0.8 large responsiveness [43]. According to Norman et al. [44], a value of half the SD of the mean change in score was used as a conservative estimate of the MIC.

#### Anchor-based approach

The diagnostic performance including calculation of MIC values of the ATRS-NL for detecting improvement was assessed by constructing receiver operating curves (ROC) [45] to evaluate the change in ATRS-NL scores as having *improved* based on GRoC and the EQ-5D-5L subdivisions *mobility* and *usual activities*. For all ROCs, the point on the curve nearest the upper-left corner was selected as the cut-off score for the MIC to minimize the sum of the percentage of patients being misclassified ((1 − sensitivity) + (1 − specificity)) [46, 47]. This point was determined by drawing a diagonal line from the upper-left corner of the ROC to the lower-right corner. The coordinate closest to this line or at which this line intersects the curve is considered to be the point closest to the upper-left corner [48] and, in this case, reflects the MIC of the ATRS-NL. The diagnostic performance of this MIC value was evaluated: sensitivity, specificity, and positive and negative predictive values (PPV/NPV) were calculated. Additionally, the percentage of patients misclassified as *improved* on the anchor using the MIC was calculated. Responsiveness was further assessed by determining the area under the ROC (AUC). AUC values > 0.5 were interpreted as the ATRS-NL having some discriminating ability concerning improvement in an anchor: 0.6–0.7 sufficient, 0.7–0.8 good, 0.8–0.9 very good and > 0.9 outstanding [49].

#### Sample size

There is no general agreement on the appropriate sample size for PROM evaluations [10]. Previous studies that provided distribution-based responsiveness data of the English ATRS included 49 [17] and 64 [27] patients. The COSMIN initiative recommends a sample size of at least 30 and preferably more than 50 participants for responsiveness evaluations [50], and this study’s sample of 47 patients is therefore considered sufficient.

## Results

### Study population

A total of 47 patients were available for follow-up at both 3 months (T1) and 6 months (T2). Patient characteristics including primary treatment are presented in Table 1. In terms of complications, there were three re-ruptures, one deep vein thrombosis and two infections that occurred after primary treatment (surgical/non-surgical). One re-rupture occurred between T2 and T1 in a non-surgically treated patient, who was subsequently treated surgically.

**Table 1:** Baseline characteristics

Characteristic	Participants* (*N* = 47)
Age (years)	41.6 (11.7)
Gender (male/female)	31/16
BMI (kg/m^2^)	25.4 (3.2)
Physically active (yes/no)	39/8
Surgical/non-surgical treatment	13/34

*Presented as mean (SD) or count

### Distribution-based approach

Data on the ATRS-NL and ES and SRM values are presented in Table 2. As expected, the ATRS-NL did not display any floor or ceiling effects, as no participant achieved the minimum or maximum score at T1 or T2.

**Table 2:** Mean ATRS-NL scores at follow-up and distribution-based statistics for entire sample (*n* = 47)

T1, mean (SD)	65.5 (15.4)
T2, mean (SD)	41.2 (21.8)
T1–T2, mean (SD)	24.4 (20.4)
ES	1.58
SRM	1.19

The ES (1.58) indicated a large change in ATRS-NL score between T2 and T1, and the SRM (1.19) a large responsiveness of the ATRS-NL for the entire study population. Using the criterion of half of the SD of the change in ATRS-NL, the estimated MIC is 10.

### Anchor-based approach

The scores on the anchors are presented in Table 3. For each anchor, two participants (4%) reported ‘more/much more impairment’ on GRoC, decreased mobility on EQ-5D-5L or decreased usual activity functioning on EQ-5D-5L, and were excluded from the anchor-based MIC analyses.

**Table 3:** Anchor outcomes

Anchor	Dimension	T1, *n* (%)	T2, *n* (%)	*p* value
GRoC	− 2	N/A	0	
	− 1		2 (4%)	
	0*		2 (4%)	
	+ 1*		18 (38%)	
	+ 2		25 (53%)	
EQ-5D-5L mobility	Level 1	2 (4%)	26 (55%)	< 0.0001
	Level 2	22 (47%)	13 (27%)	
	Level 3	19 (40%)	8 (17%)	
	Level 4	2 (4%)	0	
	Level 5	2 (4%)	0	
EQ-5D-5L usual activities	Level 1	10 (21%)	32 (68%)	< 0.0001
	Level 2	17 (36%)	11 (23%)	
	Level 3	19 (40%)	4 (9%)	
	Level 4	1 (2%)	0	
	Level 5	0	0	

*Categories that together form the category *unchanged*

Data on the ATRS-NL for patients categorized as *improved* and *unchanged* based on the GRoC and EQ-5D-5L subdivisions *mobility* and *usual activities* are presented in Table 4.

**Table 4:** ATRS-NL data by transition category on GRC and EQ-5D-5L subdivisions *mobility* and *usual activities* (*n* = 45)

Anchor	Transition	*N*	T1, mean (SD)	T2, mean (SD)	T2–T1, mean (SD)
GRoC	Improved	25	63.3 (14.2)	32.2 (18.1)	− 32.0 (19.3)
	Unchanged	20	68.5 (15.5)	50.8 (21.9)	− 18.0 (17.6)
EQ-5D-5L mobility	Improved	35	67.1 (14.0)	39.1 (21.3)	− 28.3 (21.0)
	Unchanged	10	58.5 (20.0)	44.6 (23.4)	− 13.9 (14.0)
EQ-5D-5L usual activities	Improved	29	65.5 (15.8)	40.8 (21.6)	− 25.1 (22.3)
	Unchanged	16	65.0 (15.9)	38.9 (22.0)	− 26.1 (16.2)

#### MIC estimation for improvement

The results of the ROC analysis for determining MIC cut-off using the three patient-reported anchors are presented in Table 5. The MIC ranged from 13.5 for improvement using EQ-5D-5L *mobility* to 28.5 using the GRoC. The AUC showed good (≥ 0.7) discriminating ability of the ATRS-NL in detecting improvement on the GRoC and the subdivision *mobility* of the EQ-5D-5L, and poor (0.49) discriminating ability for detecting improvement of the EQ-5D-5L subdivision *usual activities*. The results show that the calculated MIC scores misclassify less than 25% of all patients as *improved*.

**Table 5:** Responsiveness measures and MIC values for improvement on the specific anchors (*n* = 45)

Anchor	MIC^a^	AUC^b^ (95% CI)	Sensitivity (%)	Specificity (%)	PPV^c^ (%)	NPV^d^ (%)	Misclassification percentage (%)
GRoC	− 28.5	0.71 (0.56; 0.87)	60	80	79	62	21
EQ-5D-5L mobility	− 13.5	0.72 (0.56; 0.88)	77	60	87	44	13
EQ-5D-5L usual activities	− 25.5	0.49 (0.31; 0.66)	55	69	76	46	24

^a^Minimally important change

^b^Area under the ROC curve

^c^Positive predictive value

^d^Negative predictive value

## Discussion

The most important finding of this study is that the ATRS-NL is a responsive instrument capable of detecting relevant change between 3 and 6 months after ATR. This has been the first study to provide MIC data on the ATRS questionnaire in any language. There was a large range of MIC values depending on the approach used and anchor applied. Use of this injury-specific PROM can be recommended both for longitudinal research and for clinicians in the follow-up of Dutch ATR patients.

This study adheres to the requirements of the COSMIN checklist [10, 50]. In the absence of a single gold-standard external criterion multiple ones were used in a longitudinal design, and as expected most participants evidenced improvement. The distribution-based approach showed the ATRS-NL is responsive in detecting improvement at a group level. The latter was performed in a sample of 47 patients, which is considered adequate for PROM evaluations, especially given that SRM/ES data is independent of sample size [41]. The MIC values provide evidence for the use of the ATRS-NL by clinicians and researchers in selecting the group of patients who are improving. These MIC values can be applied in individual analyses too, as it is argued that similar MIC values are found for groups and individuals, albeit with a higher degree of uncertainty at the individual level [31].

Good responsiveness of the ATRS-NL was determined with the distribution-based approach. As expected, there was a large change in scores for the entire study population between 3 and 6 months after ATR, shown by the ES (1.58) and SRM (1.19) values. The few prior studies that also assessed the responsiveness of the ATRS used only distribution-based approaches and found similar large ES values (1.01 and 0.93) in the first 6 months after ATR [17, 18].

With the anchor-based approach the ability of the ATRS-NL to discriminate between patients who retrospectively report much less impairment (MIC: 28.5) and who prospectively report improved mobility (MIC: 13.5) was confirmed. Because of the lack of a gold-standard anchor for improvement after ATR, the MIC of the change in ATRS-NL scores was calculated using three patient-reported external criteria (anchors). There was a wide range in calculated MIC values with this approach, depending on which anchor was used to assess improvement. Overall, the ATRS-NL proved to be accurate in classifying patients as improved on GRoC and the EQ-5D-5L *mobility* subdivision (AUC > 0.7) and showed a low percentage (13% and 21%) of misclassifying patients as improved based on the optimal cut-off MIC. Nevertheless, the corresponding MIC values showed that relatively large changes in ATRS-NL scores are required to accurately classify patients as improved, whereas the distribution-based data showed that these large changes were occurring in this study population. The latter can be explained by the follow-up period in the recovery phase, where improvement can be expected. This period of follow-up resulted in the relatively large sample who were considered *improved* or *unchanged* and were thus available for anchor-based MIC analyses (*n* = 45). It is recommended that future studies also assess the responsiveness of the ATRS-NL in a more long-term follow-up, where relatively smaller changes in improvement can be expected.

It has been proposed that the observed MIC is smallest for the anchor that shows the highest correlation with the scale used in the study [51]. For the ATRS-NL, this concerned determining improvement in mobility on EQ-5D-5L (− 13.5). This can be explained by the original purpose of developing the ATRS: to reflect the restrictions caused by symptoms during various *physical* activities after ATR [26]. The ATRS-NL was not responsive to improvement when using the EQ-5D-5L item *usual activities*. By using this anchor a large MIC was found (− 25.5), but a small AUC (0.49) and low sensitivity and specificity (55 and 69%, respectively). This finding contrasts with those of Kearney et al. [27], who found similar correlations between the English ATRS and the EQ-5D-3L items *usual activities* and *mobility*. This is thought to be the result of using the 3-point Likert scale EQ-5D; in the present study the updated 5-point scale was used, allowing for more sensitive reporting in categorizing patients as having improved [52].

The study population consisted of both non-surgical and surgically treated patients, which we believe does not influence the results, given that short-term ATRS scores are not significantly different between treatment groups [53]. Nevertheless, only 10% of studies comparing ATR treatment methods use the ATRS as an outcome measure in comparing short-term results [53]. On the other hand, all studies comparing surgical and non-surgical treatment report re-rupture rates [53]. A limitation of this outcome measure is that it does not adequately represent the patient function. Given the discriminating ability of the ATRS-NL in detecting improvement, we suggest this practice be re-evaluated and additional well-designed trials and observational studies are performed.

Up to 50 region-specific PROMs for foot and ankle disorders are available and have been studied for their clinimetric properties [11, 12]. Similar to the studies on the ATRS questionnaire, research tends to focus on the validity and reliability of these PROMs; data on responsiveness is mostly lacking [11]. To our knowledge, of these 50 PROMs only evidence for the responsiveness of the foot and ankle ability measure (FAAM), Manchester-Oxford foot questionnaire (MOXFX), foot and ankle outcome score (FAOS) and Oxford ankle foot questionnaire for children exists (OxAFQ) [11, 12, 54]. Additionally, all of the Dutch foot and ankle PROMs show either no data on responsiveness or poor responsiveness [55], which may explain why only 6% of Dutch surgeons treating ATRs use PROMs in monitoring treatment progress after ATR [56]. It is, therefore, recommended that researchers and clinicians use the ATRS(-NL) as opposed to other foot and ankle PROMs in the follow-up of ATR patients. The lack of responsiveness reporting contrasts with the recommendations made by the COSMIN expert panel, who state it to be an essential measurement property [10]. Hence it is recommended that in future research on the development and validation of foot and ankle PROMs responsiveness also be analyzed, preferably using distribution-based and anchor-based methods.

This study has several strengths. First of all, both distribution and anchor-based methods were used to evaluate responsiveness. Because no single valid gold-standard external criterion exists and the validity of retrospective global rating of change is debated [57], this study used three different patient-reported anchors, both retrospective and prospective, as recommended by the COSMIN initiative [2, 10]. Nevertheless, no single individual external criterion is clearly valid, which is a limitation of this study. Data were gathered prospectively during a multicentre cohort study, increasing the generalizability of results. Also, this study focused on a follow-up period that is considered relevant for clinicians and researchers alike (3 and 6 months post-ATR). This is when clinicians see patients for follow-up after primary treatment and early into rehabilitation (3 months), and before return to normal function (6 months). However, the focus on an identical follow-up period in the recovery phase is another limitation of this study. In the absence of complications most patients will have improved, limiting the sample of participants reporting deterioration of symptoms and physical activity following ATR. The design and follow-up period of this study (3 and 6 months after ATR) resulted in the anticipated large improvement in ATRS-NL scores. Data on the ability of the ATRS-NL to detect regression are therefore lacking. Additionally, whether these data can be extrapolated to changes seen more in the long term—at the time of return to sport/usual function—might be debatable and would require assessing responsiveness using different, sport-specific anchors. Responsiveness at a later stage in ATR recovery should be assessed in future research, especially given that deficits persist for years [58] and return to sports is a tedious process [59].

This study provides evidence to support the use of the ATRS(-NL) by clinicians and researchers in the follow-up of ATR patients. The ATRS-NL is valid, reliable, easy to administer and score and—as shown by the current study—responsive to change in the clinical follow-up period (3 and 6 months) after ATR. In addition, MIC values have been derived from this study. De Vet et al. have already acknowledged that no universal MIC value for a single PROM exists [47]. It is, therefore, advised that a single value be set, but with a small range to allow for variation in interpretation [47, 60]. It is recommended researchers and clinicians use a MIC of 13.5 as the minimum change in score to consider patients as having improved, as the results show this value to accurately detect prospective improvement on EQ-5D-5L mobility. To identify the subgroup of patients who show the best improvement between 3 and 6 months after ATR, we suggest a MIC of 28.5, as this is the cut-off value for detecting patients who retrospectively report ‘much less impairment’ on GRoC.

## Conclusion

The position of the ATRS-NL as a primary outcome measure in longitudinal research and clinical practice is confirmed: it is a valuable tool in investigating the efficacy and effectiveness of an intervention. MIC values of 13.5 and 28.5 are recommended to consider patients as having improved and greatly improved between 3 and 6 months after ATR. Overall, we believe this study is an important step in value-based healthcare by contributing towards more valid, reliable and responsive PROMs.
